# Visual Cortex Inspired CNN Model for Feature Construction in Text Analysis

**DOI:** 10.3389/fncom.2016.00064

**Published:** 2016-07-14

**Authors:** Hongping Fu, Zhendong Niu, Chunxia Zhang, Jing Ma, Jie Chen

**Affiliations:** ^1^School of Computer Science and Technology, Beijing Institute of TechnologyBeijing, China; ^2^School of Software, Beijing Institute of TechnologyBeijing, China

**Keywords:** convolutional neural networks, biologically inspired feature construction, feature encoding, answer recommendation, community question answering, text analysis

## Abstract

Recently, biologically inspired models are gradually proposed to solve the problem in text analysis. Convolutional neural networks (CNN) are hierarchical artificial neural networks, which include a various of multilayer perceptrons. According to biological research, CNN can be improved by bringing in the attention modulation and memory processing of primate visual cortex. In this paper, we employ the above properties of primate visual cortex to improve CNN and propose a biological-mechanism-driven-feature-construction based answer recommendation method (BMFC-ARM), which is used to recommend the best answer for the corresponding given questions in community question answering. BMFC-ARM is an improved CNN with four channels respectively representing questions, answers, asker information and answerer information, and mainly contains two stages: biological mechanism driven feature construction (BMFC) and answer ranking. BMFC imitates the attention modulation property by introducing the asker information and answerer information of given questions and the similarity between them, and imitates the memory processing property through bringing in the user reputation information for answerers. Then the feature vector for answer ranking is constructed by fusing the asker-answerer similarities, answerer's reputation and the corresponding vectors of question, answer, asker, and answerer. Finally, the Softmax is used at the stage of answer ranking to get best answers by the feature vector. The experimental results of answer recommendation on the Stackexchange dataset show that BMFC-ARM exhibits better performance.

## 1. Introduction

Community Question Answering (CQA) has attracted a lot of attentions from both research and industry communities in recent years. A fundamental problem in CQA is answer recommendation, which recommends the best answer of a question to the asker who posts the question.

Most previous research takes this problem as a ranking task and employs learning-to-rank algorithms to rank answers. Then the answer in the top of the answer list is recommended to users. To achieve this, most researchers focus on constructing complex and novel features (e.g., lexical features, syntactic features, and semantic features) to improve the recommendation performance. For example, Surdeanu et al. (2011) use linguistically motivated features to rank answers to non-factoid questions. They exploit natural language processing such as named-entity identification, syntactic parsing, and semantic role labeling to construct similarity features, translation features, density features, and frequency features.

However, feature construction is a time-consuming and labor-consuming problem which needs huge priori knowledge and experience, especially with the increasingly huge amount of questions and corresponding answers in CQA. Nowadays, many researchers focus on constructing features automatically using neural network. They only focus on the information of questions and answers, which is not suitable for CQA containing huge social information. It is worth to leverage the social information presented in CQA that users tend to focus or vote answers according to the relation with others.

Since, biologically inspired models are gradually proposed to solve the problem in text analysis recently and the biological research of primate visual cortex, traditional CNN can be improved by introducing attention modulation and memory processing of primate visual cortex. In this paper, we employ the attention modulation and memory processing of primate visual cortex to enhance the CNN model, and propose a biological-mechanism-driven-feature-construction based answer recommendation method (BMFC-ARM) to recommend the best answer for given questions in community question answering. In order to support feature construction, BMFC-ARM imitates the attention modulation property by introducing the asker-answerer information of given questions and computing the similarity between them, and then brings in the user reputation information of users who have answered the questions, which imitates the memory processing property. After feature construction, the Softmax is used at the stage of answer ranking to get the best answer. The experimental results of answer recommendation on the Stackexchange dataset show that the BMFC-ARM exhibits better performance.

The rest of this paper is organized as follows. Section 2 describes the related work. The proposed BMFC-ARM is introduced in Section 3, which contains biological mechanism driven feature construction and answer ranking. Section 4 gives details of experiments and corresponding results. Finally, Section 5 summarizes conclusion and future work.

## 2. Related work

### 2.1. Answer recommendation

Answer recommendation is the basis research in CQA, which is designed to recommend the best answer to users. Wang et al. (2009) and Tu et al. (2009) proposed an analogical reasoning-based method to model question-answer relations to rank answers. Hieber and Riezler (2011) focused on the challenge of identifying high quality content caused by the inherent noisiness of user generated data. They proposed a series of features to model answer quality and expended the query, then used perceptron and Ranking SVM to rank answers. To recommend a reasonable answer to users, Liu et al. (2014) recognized questions in microblog and used collaborative filtering methods with integrated standard features and contextual features which are extracted from auxiliary resources. Beyond textural features used in previous works, user information is also investigated in answer ranking. Zhou et al. (2012) took advantage of three kinds of user ptofile information: level-related, engagement-related, and authority-related, and employed SVMRank and ListNet for ranking answers. To avoid the manual quality control mechanisms, Dalip et al. (2013) proposed a learning to rank approach, and used textual and non-textual features which can represent the quality of query and answer pairs to rank answers. Specifically, the non-textual features contain user, review, and user-graph features.

### 2.2. Deep learning for text analysis

Kalchbrenner et al. (2014) proposed a dynamic convolutional neural network with a dynamic k-Max pooling to model sentences. Hu et al. (2015) adapted the convolutional strategy in vision and speech, and then proposed convolutional neural network models for matching two sentences.

In classification tasks, Wang et al. (2016) proposed a framework to expand short texts based on word embedding clustering and convolutional neural network to overcome the worse classification performance caused by data sparsity and semantic sensitivity. Lai et al. (2015) introduce a recurrent neural network for text classification. Zeng et al. (2014) exploit convolutional neural network to extract word level features and sentence level features. Santos et al. (2015) proposed a a new pairwise ranking loss function and used convolutional neural network for relation classification.

Deep learning has been proven to be effective for many text analysis tasks. Recently some researchers brought deep learning into question answering. Bordes et al. (2014a,b) used an embedding model to project question-answer pairs into a joint space. tau Yih et al. (2014) used convolutional neural network to measure the similarity of entity and relation of a question with those in knowledge base for single-relation question answering. Iyyer et al. (2014) introduced a recursive neural network to model textual composition for factoid question answering. Zhou et al. (2016) aimed to find answers of previous queries to new queries, and used neural network architecture to learn the semantic representation of queries and answers in community question answering retrieval.

Different from previous deep learning methods only focusing on the semantics of question-answer pairs to rank answers, we also take user information into account in this paper, which is an very important aspect in community question answering.

## 3. Methodology

In this section, we present the proposed approach BMFC-ARM, which contains biological mechanism driven feature construction (BMFC) and answer ranking. First, an overview of the framework of BMFC-ARM is given. Then we describe the BMFC method and answer ranking in detail.

### 3.1. Overview of BMFC-ARM

Answer recommendation can be viewed as a ranking problem. Given a set of questions *Q* in a community question answering (CQA) system, each question *q*_*i*_ ∈ *Q* contains a list of answers *A*_*i*_ = {*a*_*i*1_, *a*_*i*2_, …, *a*_*ib*_, …, *a*_*in*_}, where *a*_*ib*_ is the best answer selected by asker or CQA systems, our goal is to learn a ranker according to these question-answer pairs, then recommend the best answer to any additional questions.

**Figure 1:**
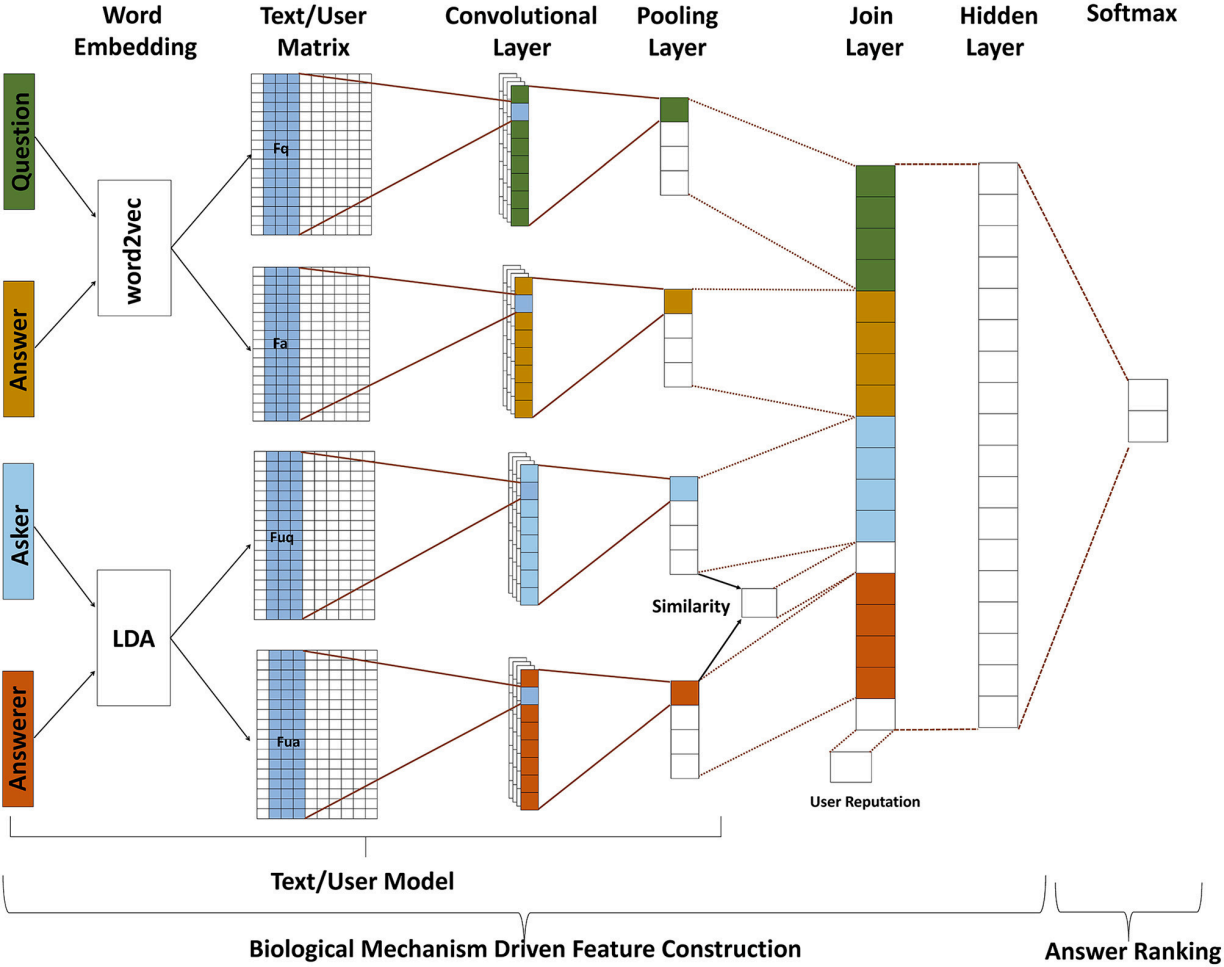
**The framework of BMFC-ARM, which contains two stages: BMFC and answer ranking**. BMFC method is to automatically construct features by introducing the attention modulation and memory processing, which contains three parts: text model, user model, and feature fusion. The feature representation of questions, answers, and users are obtained from text model and user model respectively, and then the feature fusion constructs all feature representations together with the similarity of asker-answerer pairs and answerer's reputation into a combined feature vector. At last, Softmax is implemented to rank answers and recommend the best answer accordingly.

The proposed BMFC-ARM consists of two stages: BMFC and answer ranking which shown in Figure 1. BMFC method is to automatically construct features by introducing the attention modulation and memory processing, which contains three parts: text model, user model, and feature fusion. First, questions and their corresponding answers are passed through text model to get their feature vectors which contain semantic information. At the same time, the corresponding asker information and answerer information are passed through user model to get their feature vectors. In order to introduce the attention modulation and memory processing properties, BMFC imitates the attention modulation property by introducing the asker information and answerer information of given questions through user model and computing the similarity between them, and then brings in the user reputation information of user who answered the questions, which imitates the memory processing property. After getting the feature representation of questions, answers, askers and answerers, feature fusion is used to combine those features into a single vector. After feature construction, answer ranking employs Softmax to recommend the best answer.

### 3.2. Biological mechanism driven feature construction (BMFC)

For the openness of CQA, all users can answer questions, which results in the unstable quality of answers. For the sociality of CQA, users get more interaction with each other when they are similar, and may select the answer that provided by the answerer who is similar with them as the best answer. Therefore, in this paper, we assume that when users choose an answer as the best answer in CQA, their thinking process have two properties: (1) whether the answer is related to the question; (2) whether the answerer is the person they care about or familiar with.

**Figure 2:**
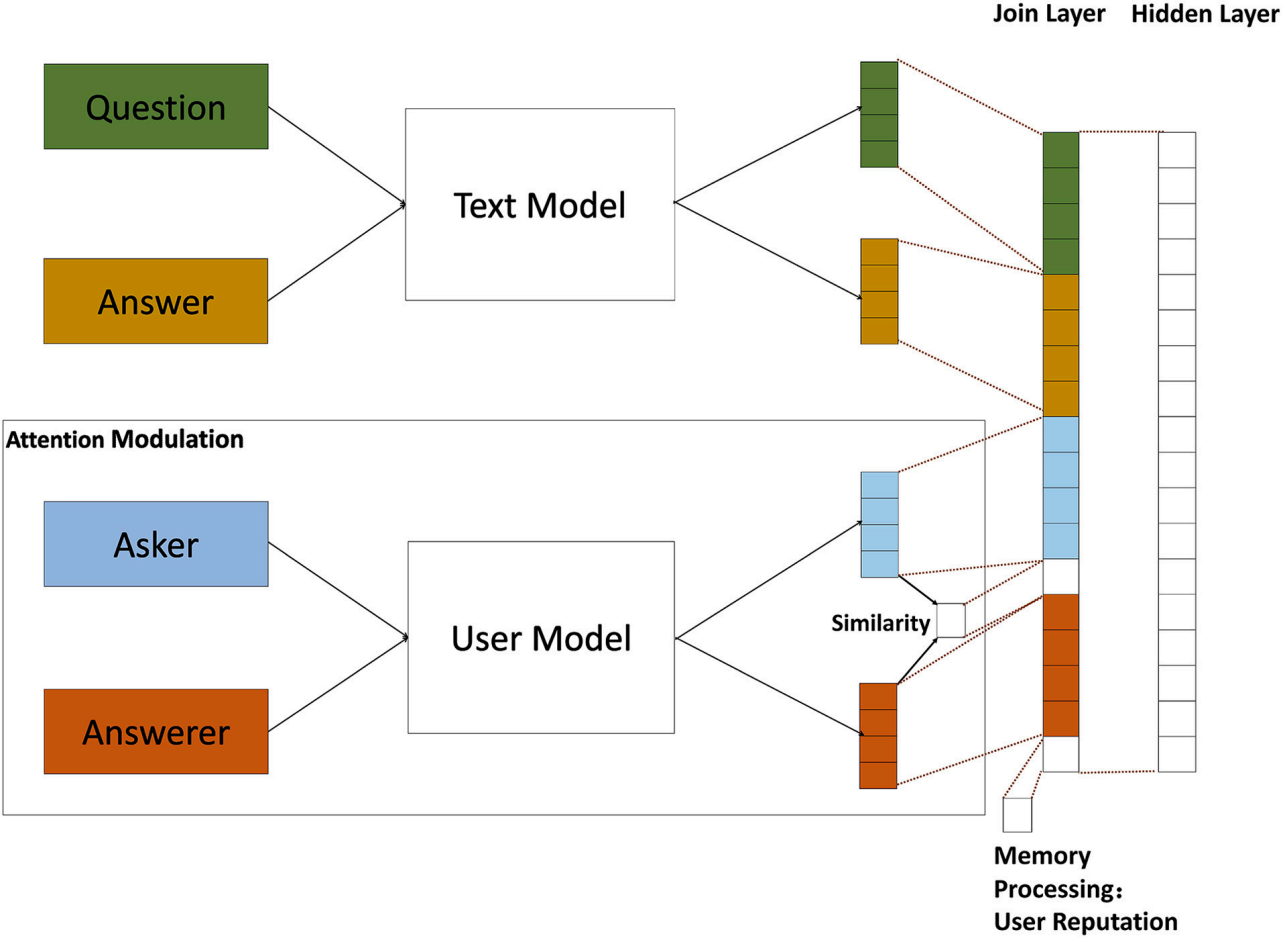
**The BMFC method, which contains three parts: text model, user model, and feature fusion**. First, questions and their corresponding answers are passed through text model to get their feature vectors which contain semantic information. At the same time, the corresponding asker and answerer are passed through user model to get user feature vector. In order to introduce the attention modulation and memory processing properties, BMFC imitate the attention modulation property by introducing the asker-answerer information of given questions through user model and computing the similarity between them, and then bring in the user reputation information of user who answered the questions, which imitates the memory processing property. After getting the feature representation of questions, answers and users, feature fusion is used to combine those features into a single vector.

According to the assumption, we introduce attention modulation and memory processing of primate visual cortex, and propose a biological mechanism driven feature construction (BMFC) method. As users may choose an answer which answered by the person similar to them as the best answer, BMFC imitate the attention modulation property by computing the similarity between askers and answerers of given questions based on user model to reflect the relation between askers and answerers. The reputation information represents the quality of answers user answered. In order to reflect the relevance of answers and questions, BMFC method introduces user reputation to imitate the the memory processing property. BMFC method contains text model, user model and feature fusion. The flow of BMFC method is shown in Figure 2.

#### 3.2.1. Text model

**Figure 3:**
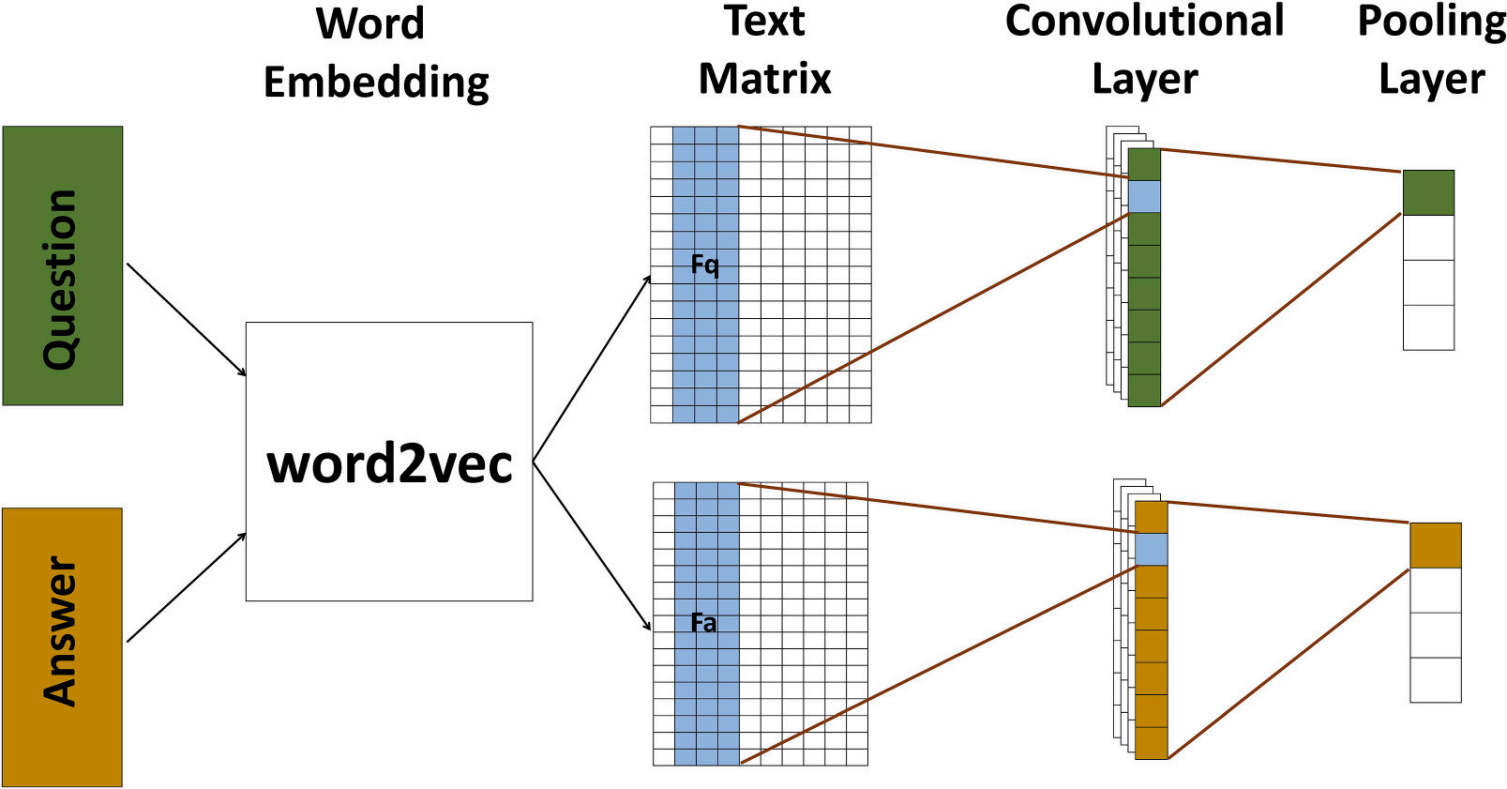
**The text model is used to map text into its corresponding feature representions**. We use word2vec to tranform texts into vectors, and then use two channel convolutional neural network to model questions and answers. All texts pass through a convolutional layer followed by a pooling layer. After text model, texts are presented by their corresponding features.

The text model in BMFC is based on convolutional neural network which is shown in Figure 3. It contains two channels to model question and answer respectively, and each channel contains a convolution layer followed by a simple pooling layer.

##### 3.2.1.1. Text matrix

Our text model first transforms the original text into vectors. Inspired by Kalchbrenner et al. (2014), we use word2vec that takes advantage of the context of the word which contains more semantic information to do the word embedding for each word in a text, and then construct the text matrix ***T*** ∈ ***R***^*d* × |*t*|^ shown bellow:

$$\begin{array}{l} T=\left[\begin{array}{ccccc} | & | & | & | & |\\ w_{1} & \cdots & w_{i} & \cdots & w_{\left|t\right|}\\ | & | & | & | & | \end{array}\right] \end{array}$$

where $w_{i}\in R^{d}$ is the word embedding of a word in the text which contains |*t*| words and *i* is the position of the word in the text.

Then we will give a description of convolutional layer and pooling layer used in each channel in next sections.

##### 3.2.1.2. Convolutional layer

Convolutional layer is to convolve a matrix of weights with the matrix of activations at the layer below, which has two kinds of convolution: narrow convolution and wide convolution Kalchbrenner et al. (2014). In our framework, we use wide convolution which can deal with words at boundaries, and give equal attention to words in different positions. And we use ReLU as the activation function *f*(·). Given the text matrices ***T*** ∈ ***R***^*d* × |*t*|^ and a convolution filter ***k*** ∈ ***R***^*m*^, the convolution operation between them results in a vector *c* ∈ ***R***^|*t*| + *m* −1^. Each element of **c** is computed as follows:

(1) $$\begin{array}{l} c_{i}=f\left({T_{i-m+1;i}}^{T}\cdot \text{k}+b\right) \end{array}$$

where |*t*| is the number of text word, *b* is the bias, *m* is the width of convolutional filter.

##### 3.2.1.3. Pooling layer

After convolutional layer, the input texts are represented by the extracted features, and then passed through the pooling layer. Pooling layer is used to reduce the dimension of features obtained through the convolutional layer and aggregate feature information from different parts. There are three commonly used pooling methods: average-pooling, max-pooling, and stochastic-pooling. Boureau et al. (2010) compared average-pooling and max-pooling in detail. In this paper, we use max pooling which is the most widely used pooling methods. It chooses the feature with the maximum value in an area as shown in Equation (2).

(2) $$\begin{array}{l} c_{p}=max\left\{c\right\} \end{array}$$

Then, the text matrix, convolutional layer and pooling layer form our text model which builds rich feature representations of the input question and answer.

Unlike previous works which just map the question and answer into a vector space, BMFC takes user information into account modeling asker and answerer into the same vector space, and evaluates the relatedness of asker-answerer pairs based on user model.

#### 3.2.2. User model

To introduce the attention modulation and memory processing property into BMFC, we propose user model which represents user information. In this paper, we use users' self descriptions as user information.

**Figure 4:**
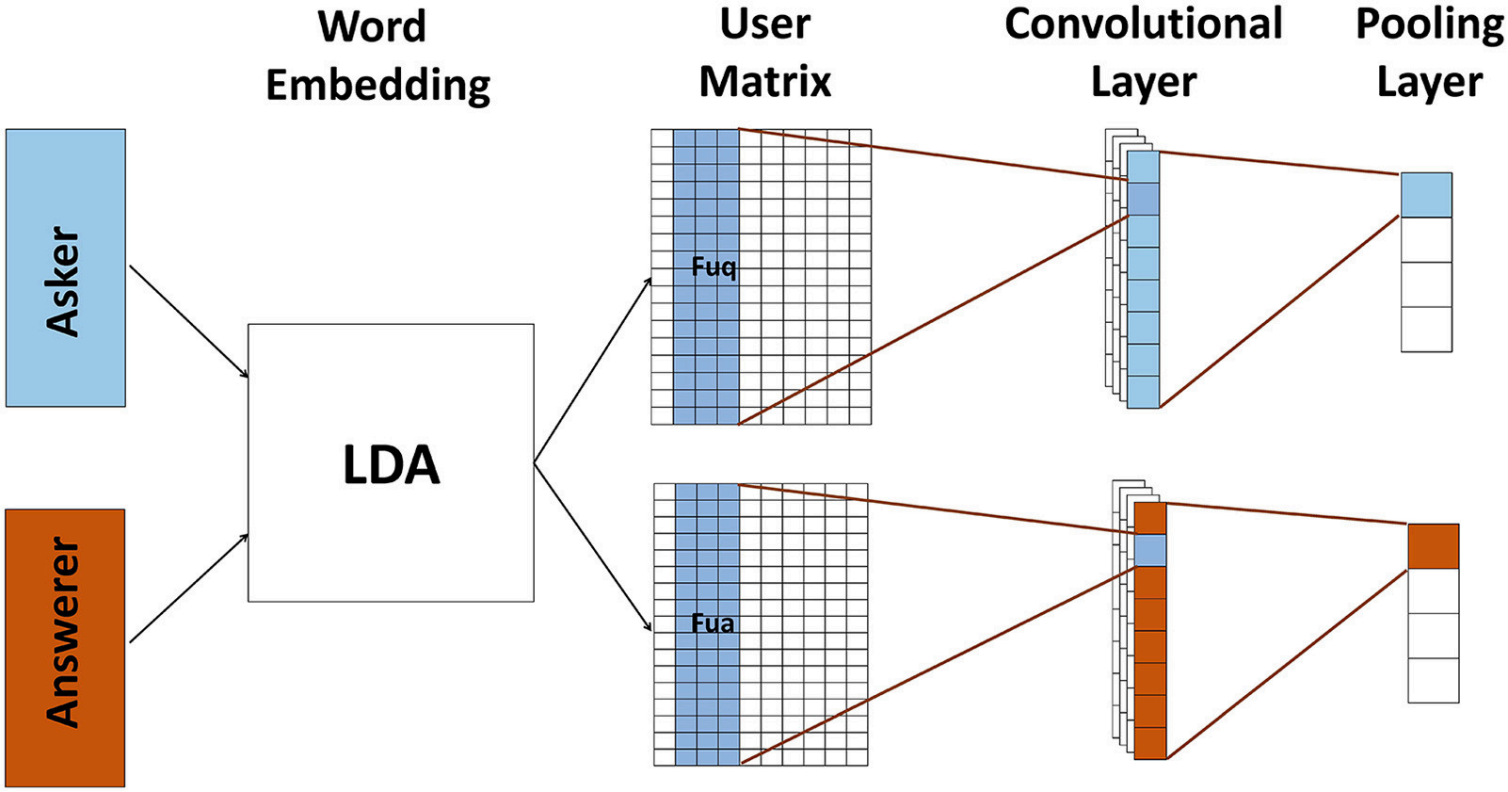
**The user model for mapping user information into its corresponding feature representation**. We use LDA to generate user matrix, and then use two channel convolutional neural network to model askers and answerers. All user matrixes pass through a convolutional layer followed by a pooling layer. After user model, askers, and answerers are presented by their corresponding features.

Same as text model, user model is based on CNN which contains two channels to represent askers and answerers, respectively. And each channel has a convolutional layer and a pooling layer shown in Figure 4. Since users' self descriptions are very short, e.g., some just contain keywords, we use Latent Dirichlet Allocation (LDA) Blei et al. (2003) to generate user matrix ***U*** ∈ ***R***^*d* × |*u*|^.

$$\begin{array}{l} U=\left[\begin{array}{ccccc} | & | & | & | & |\\ w_{u1} & \cdots & w_{ui} & \cdots & w_{u|u|}\\ | & | & | & | & | \end{array}\right] \end{array}$$

where *d* is the dimension of word vector, $w_{ui}\in R^{d}$ is the word representation of a word in user self description, |*u*| is the number of words and *i* is the position of ***w***_*ui*_.

The convolutional layer and pooling layer in user model are similar with those in text model.

#### 3.2.3. Feature fusion

After text model and user model, the information of questions, answers, and corresponding askers and answerers is represented by numeric vectors ***v***_*q*_, ***v***_*a*_, ***v***_*uq*_, and ***v***_*ua*_, respectively. Then, we compute the similarity between asker and answerer to represent their relations. Here, we use cosine similarity shown as follows:

(3) $$\begin{array}{r} s_{uqua}=\frac{\upsilon_{uq}\cdot \upsilon_{ua}}{\left||\upsilon_{uq}||\times ||\upsilon_{ua}|\right|} \end{array}$$

where *s*_*uqua*_ is the similarity between asker and answerer, ||**υ**_*uq*_|| is the Euclidean norm of **υ**_*uq*_ = υ_*uq*1_, υ_*uq*2_, ⋯ , υ_*uqn*_ defined as $\sqrt{\upsilon_{uq1}^{2}+\upsilon_{uq2}^{2}+\cdots +\upsilon_{uqn}^{2}}$. Similarly, ||**υ**_*ua*_|| is the Euclidean norm of **υ**_*ua*_.

Then, BMFC method concatenates the asker-answerer similarities *s*_*uqua*_, answerer's reputation *v*_*r*_, and corresponding vectors of question, answer, asker, and answerer into a single vector which can be represented as $\upsilon =\left[{\upsilon_{q}}^{T};{\upsilon_{a}}^{T};{\upsilon_{uq}}^{T};s_{uqua};{\upsilon_{ua}}^{T};v_{r}\right]$. Then, BMFC uses a hidden layer to interact the different parts of **υ** to construct the final feature to represent samples:

$$\begin{array}{l} \phi \left(\text{w}\cdot \upsilon +b\right) \end{array}$$

where ***w*** is the weight vector of the hidden layer, *b* is the bias, and ϕ(·) is the *tanh* function.

### 3.3. Answer ranking

After feature construction using BMFC method, question-answer pairs, and their corresponding users' information are represented through a vector **V**. In our method, we use a simple pointwise ranking method to rank answers. Softmax is often used in classification problem, which gives a probability of the sample belongs to each class. Given the sample vector **V**, the probability that it belongs to class *j* (*j* = 1, …, *K*) is computed by Equation (4). Then, answers are ranked according to this probability.

(4) $$P(y=j|V)=\frac{e^{V^{T}W_{j}}}{\sum_{k\;=\;1}^{K}e^{V^{T}W_{k}}}$$

where **W**_*k*_ is the weight vector of the *k*th class.

## 4. Experiment

### 4.1. Experiment setting

#### 4.1.1. Dataset

In our experiments, the raw data we use are from Stack Exchange Data Dump^1^, which is an anonymized dump of all user-contributed content on the Stack Exchange network^2^. The dataset contains 238 sites and each site consists of questions and corresponding answers of each question. We select around 840 resolved questions in four sites: movies, sports, travel, and music.

We split the dataset of 2385 question-answer pairs into a training set (train, 80%), a development set (dev, 10%), and a testing set (text, 10%) by randomly selecting 669 questions for training set, 87 questions for development set, and 84 questions for testing set, which are shown in Table 1. Here, each pair of question and its answer together with the corresponding asker and answerer constitutes an example. The example with best answer is considered as the most relevant example among all examples with other answers of the same question. This setup is used in training set, development set, and testing set.

**Table 1:** **Summary of the answer recommendation dataset**.

**Data**	**# Questions**	**# QA Pairs**	**# Askers**	**# Answerers**	**# Users**
Train	669	1908	363	597	787
Dev	87	239	71	160	209
Test	84	238	76	173	229
Total	840	2385	428	689	912

*#Question, #QA pairs, #Askers, and #Answerers are the number of questions, question-answer pairs, askers, and answerers respectively. #Users is the total number of askers and answerers except the overlap of them*.

#### 4.1.2. Word embeddings

In our experiments, we use word2vec^3^ to get word embeddings for questions and answers in text model, while using LDA to generate user representation for askers and answerers in user model.

For text model which represents question and answer information, we use word2vec to get word embeddings, which contains more semantic information by making use of the context of words. Similar with Kim (2014), Yu et al. (2014), we use the fixed word embeddings trained on all sites of Stack Exchange Data Dump. And we use the skipgram model with window size 5 to train word embeddings. Then words are represented by 50-dimensional vectors.

Due to the brief self description of users, we use JGibbLDA^4^ trained by Gibbs sampling to generate word embeddings for the user model. The parameter α is set as 0.5, β is set as 0.1, topic number is set as 100, and each topic contains 50 words.

#### 4.1.3. Parameters

The width *m*_*t*_ of convolutional filter of the text model is set to 5, and the width *m*_*u*_ of the user model is set to 2 according to experimental results. The convolutional maps of both models are 100, and the depth of the convolutional filter is set to 50 which is equal to the dimension of word vectors. We use ReLU as the activation function and max pooling method.

Similary with Kim (2014), we use stochastic gradient descent over mini-batches to train the BMFC-ARM where batch size is set to 50.

#### 4.1.4. Evaluation

For the task of answer recommendation, top answers in ranking list determine users' satisfaction. Therefore, we use Precision@N and Mean Reciprocal Rank(MRR) as metrics to evaluate our proposed method, which consider the position factor. Both of them are commonly used in information retrieval and question answering. Since we want to recommend the best answer to users, we use Precision@1(*N* = 1) in this paper, which means that we only focus on the precision of the first answer. Then Precision@1 is set to 1 if the best answer is ranked as first, 0 otherwise.

MRR takes the position of relevant answers into consideration. Where Reciprocal Rank is the multiplicative inverse of the rank of the first correct answer, and Mean Reciprocal Rank is the average of Reciprocal Rank that taken over all questions. MRR is computed as

$$\begin{array}{l} MRR=\frac{1}{\left|Q\right|}\overset{\left|Q\right|}{\underset{q=1}{\sum }}\frac{1}{rank\left(q\right)} \end{array}$$

where |*Q*| is the number of questions in test dataset, *rank*(*q*) is the position of the best answer in the resulting answer list.

### 4.2. Results

In this section we report the results of answer recommendation obtained by BMFC-ARM, and give a comparison among different methods (CNN-1, CNN-2, CNN-4, CNN-4M, CNN-4A, and BMFC-ARM). CNN-1 method just considers the information of questions and answers, which is passed through a single CNN network to obtain the corresponding features. CNN-2 method is a CNN network with two channels, which means that question information and answer information are passed through one channel respectively, and then obtains their corresponding features. CNN-2 just considers the information of questions and answers, which is similar with CNN. CNN-4 method considers both question-answer information and user information, which means that the information of question, answer, asker and answerer is passed through four channels of CNN network respectively, and then obtains their corresponding features. Based on CNN-4, CNN-4M brings in the answerer's reputation to imitate memory processing property, and CNN-4A introduces the similarity between askers and answerers. The proposed BMFC-ARM imitates the attention modulation property by introducing the asker-answerer information of given questions and computing the similarity between them, and brings in the user reputation information for users who answered the questions to imitate the memory processing property. The details of data used in this experiment are shown in Table 1. The evaluation results measured by MRR and P@1 are reported over this random split.

When users' information is added, we compare the effects of different widths *m*_*u*_ of convolution filter due to the brief self description of users. Unlike setting 5 as the width of convolution filter in the text model of representing questions and answers, our experiments compare the user model setting 2, 3, 4, and 5 as the width of convolution filter, respectively. Table 2 gives the answer recommending results using different convolution filter widths in the user model. As seen from Table 2, when *m*_*u*_ = 2, the value of MRR and P@1 of all methods are higher than the cases of *m*_*u*_ = 3, 4, and 5. The reason behind this result may be due to the brief user information. Therefore, in the subsequent experiments of this paper, the convolution filter width of the user model is set to 2.

**Table 2:** **Results with different widths of convolutional filter in user model**.

**Model**	***m_u_* = 2**		***m_u_* = 3**		***m_u_* = 4**		***m_u_* = 5**	
	**MRR**	**P@1**	**MRR**	**P@1**	**MRR**	**P@1**	**MRR**	**P@1**
CNN-4	0.7498	0.5595	0.7403	0.5357	0.7409	0.5357	0.7240	0.5119

*The CNN-4 method considers both question-answer information and user information, which means that the information of question, answer, asker, and answerer is passed through four channels of CNN network respectively, and then obtains their corresponding features. m_u_ means the convolution filter width in user model*.

**Figure 5:**
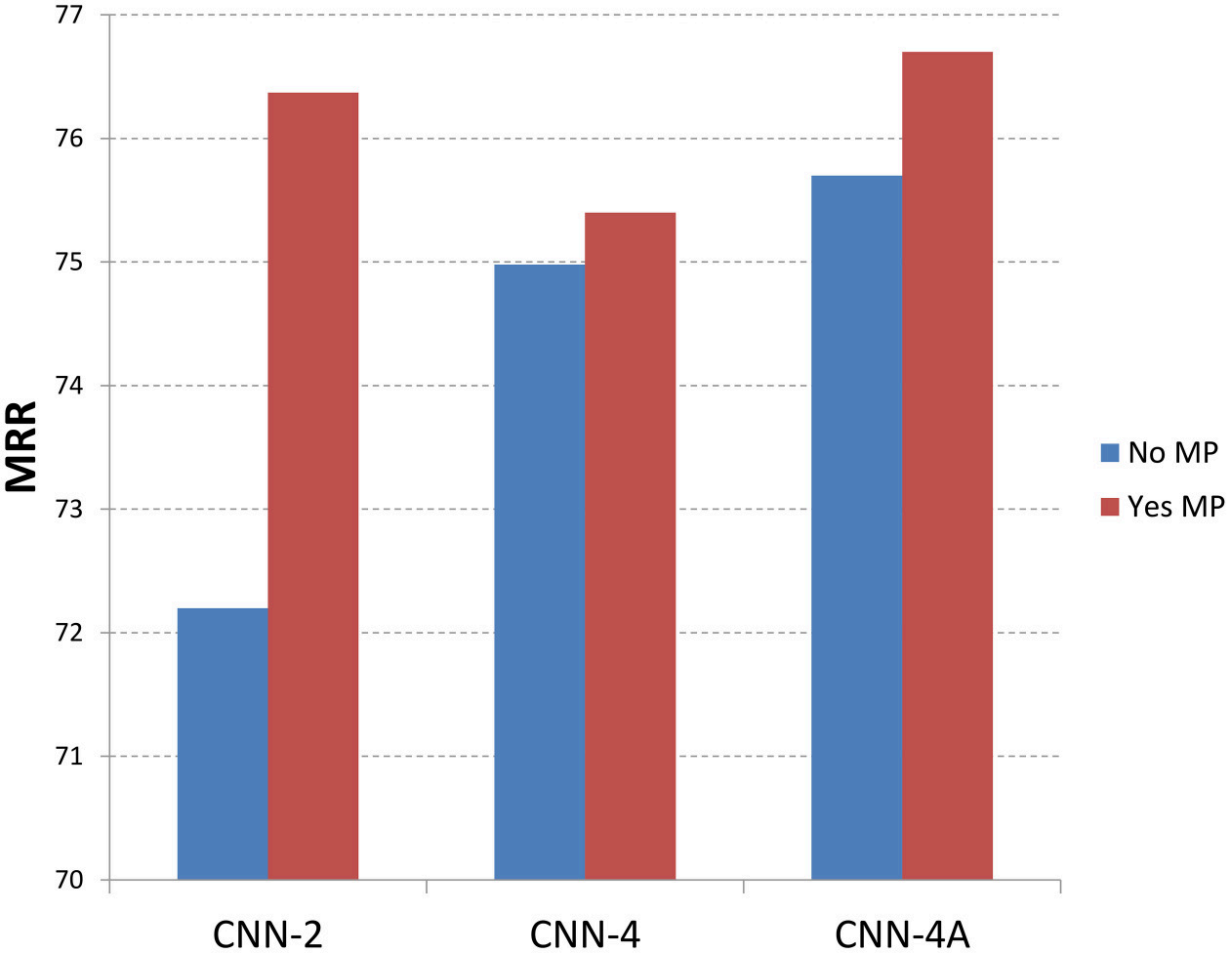
**Results with different methods along with memory property information (MRR)**. No MP means that methods do not introduce the memory property, where Yse MP means that methods considered the memory property.

**Figure 6:**
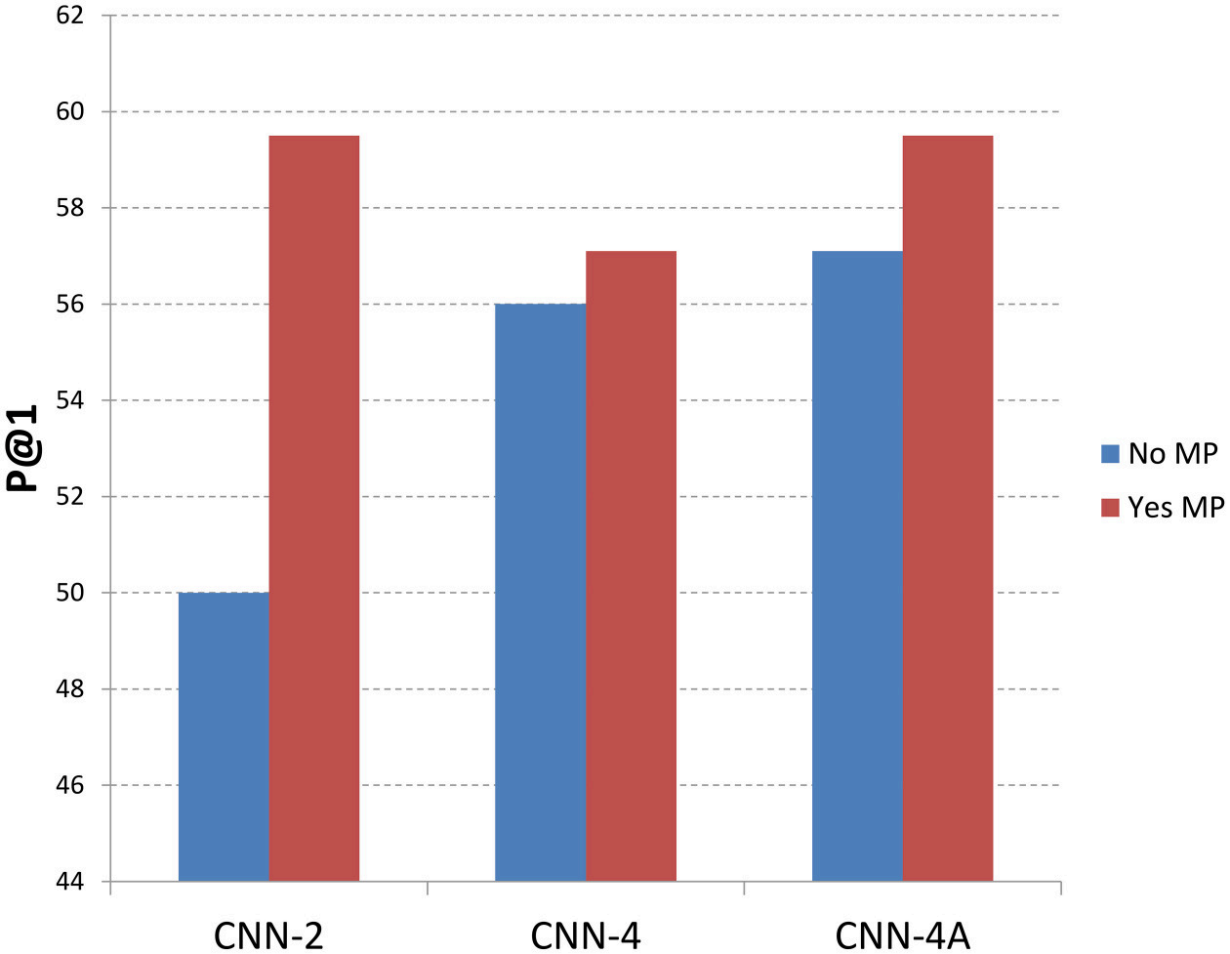
**Results with different methods along with memory property information (P@1)**. No MP means that methods do not introduce the memory property, where Yse MP means that methods considered the memory property.

Figures 5, 6 show the recommendation results with different methods with memory processing property and without memory processing property. Figure 5 shows the results with MRR measure and Figure 6 gives the P@1 measure. In these two figures, the blue histogram represents methods which do not consider the memory process mechanism, while the red histogram represents methods that considered the memory process mechanism. From Figure 5 we can see that CNN-2, CNN-4, and CNN-4A obtain better performance by introducing the memory process mechanism, which shows that the memory processing mechanism through user reputation is useful to recommend best answers. For CNN-2 which just considers question information and answer information through two channels of CNN, the recommendation result performs better through adding user reputation, which shows that memory processing mechanism plays an important role in answer recommendation. From the recommendation results shown in Figure 6 we can find that P@1 measure has the same tendency with MRR measure, which also shows that methods with memory processing mechanism get better performance than those without memory processing mechanism.

The recommendation results of different methods (BMFC-ARM, CNN-1, CNN-2, CNN-4, CNN-4M, and CNN-4A) with evaluation metrics of MRR and P@1 are shown in Table 3. From Table 3, it is promising to observe that the proposed BMFC-ARM outperforms those CNN-1, CNN-2, CNN-4, CNN-4M, and CNN-4A with MRR and P@1 measure. It is probably because that BMFC-ARM takes user information into account introducing the attention modulation property and memory processing property. From methods CNN-2 and CNN-4, we can find that CNN-4 with user information performs better than CNN-2 which just uses question and answer information. This shows the importance of user information for answer recommendation. Therefore, when recommending the best answer to users in CQA, we need to take the relation information between askers and answerers into account rather than just considering question and answer information. The phenomenon that CNN-4M performs better than CNN-4 may be caused by the introduced memory processing property. This indicates that our memory processing property is useful by introducing user reputation information. And the phenomenon that CNN-4A performs better than CNN-4 shows that recommendation results can be improved by considering the similarity between askers and answerers which brings in users' relation. The method CNN-4A outperforms CNN-2 shows that through introducing the attention modulation property, represented by user information and the similarity between askers and answers, can improve the recommendation results.

**Table 3:** **Results with different methods (CNN-1, CNN-2, CNN-4, CNN-4M, CNN-4A, and BMFC-ARM)**.

**Model**	**MRR**	**P@1**
CNN-1	0.6933	0.4524
CNN-2	0.7220	0.5000
CNN-4	0.7498	0.5595
CNN-4M	0.7540	0.5712
CNN-4A	0.7567	0.5714
BMFC-ARM (Our model)	0.7673	0.5952

*CNN-1 method just considers the information of questions and answers, which is passed through a single CNN network to obtain the features. CNN-2 method is a CNN network with two channels, which means that question information and answer information are passed through one channel respectively. CNN-4 method considers both question-answer information and user information, which means that the information of question, answer, asker, and answerer is passed through four channels of CNN network respectively. Based on CNN-4, CNN-4M brings in the answerer's reputation to imitate memory processing property, and CNN-4A introduces the similarity between askers and answerers. BMFC-ARM imitates the attention modulation property by introducing the asker-answerer information of given questions and computing the similarity between them, and brings in the user reputation information which imitates the memory processing property*.

## 5. Conclusion

Convolutional neural networks (CNN) are hierarchical artificial neutral networks, which are popularly used in natural language processing. In this paper, we propose the BMFC-ARM to recommend best answers for given questions in community question answering, which is an improved CNN by introducing attention modulation and memory processing of primate visual cortex. In order to support the feature construction, we imitate the attention modulation property by computing the similarity of asker-answerer information of given questions, and bring in the user reputation information for users who answered the questions, which imitates the memory processing property. Softmax is used at the stage of answer ranking to get the best answer. The answer recommendation experimental results on the Stackexchange dataset show that BMFC-ARM exhibits better performance.

In the future, we will investigate how to bring the users' sentiment information of questions into our framework and find a novel way to represent the text.

## Author contributions

HF prepared the methods of feature construction and answer ranking. JC provided the related researches. HF and JM conducted the experiments. HF prepared the manuscript. ZN and CZ initiated this study and supervised all aspects of the work. All authors discussed the results and commented on the manuscript.

### Conflict of interest statement

The authors declare that the research was conducted in the absence of any commercial or financial relationships that could be construed as a potential conflict of interest.

## Footnotes

^1^https://archive.org/details/stackexchange

^2^http://stackexchange.com

^3^https://code.google.com/archive/p/word2vec/

^4^http://jgibblda.sourceforge.net/
